# Sarcopenia, frailty syndrome and obstructive sleep apnea as elements of the aging phenotype in head and neck cancer and upper airway disorders: a scoping review

**DOI:** 10.3389/fmed.2026.1858903

**Published:** 2026-07-30

**Authors:** Jacek Kobak, Karolina Chmielnicka, Paulina Komorowska, Daniela Alterio, Luca Bergamaschi, Barbara A. Jereczek-Fossa, Mateusz Szczupak, Bogusław Mikaszewski

**Affiliations:** 1 Department of Otolaryngology, Faculty of Medicine, Medical University of Gdańsk, Gdańsk, Poland; 2 Department of Otolaryngology, University Medical Center, Gdańsk, Poland; 3 Department of Radiation Oncology, IEO European Institute of Oncology IRCCS, Milan, Italy; 4 Department of Oncology and Hemato-oncology, University of Milan, Milan, Italy; 5 Department of Anaesthesiology and Intensive Therapy of the Nicolaus Copernicus Hospital in Gdańsk, Gdańsk, Poland

**Keywords:** frailty syndrome, head and neck cancer, obstructive sleep apnea, otolaryngology, sarcopenia, upper airway disorders

## Abstract

**Background:**

Sarcopenia, frailty syndrome, and obstructive sleep apnea (OSA) are increasingly recognized as age-related conditions with overlapping biological pathways. Their coexistence may be relevant in head and neck cancer and upper airway disorders, but direct evidence integrating OSA with head and neck cancer (HNC) and geriatric vulnerability remains limited.

**Objective:**

This scoping review aimed to map the evidence on the relationships among sarcopenia, frailty, and OSA in the context of head and neck cancer and upper airway disorders, and to determine whether the available literature supports an integrated clinical model linking OSA-related vulnerability with HNC outcomes.

**Methods:**

The review was conducted in accordance with PRISMA ScR guidelines. A literature search was performed in PubMed, EBSCO, and Google Scholar for studies published between January 2015 and December 2025. Eligible studies included adult populations and addressed sarcopenia, frailty, OSA, or their clinically relevant relationships in head and neck cancer and upper airway disorders.

**Results:**

Twelve studies met the inclusion criteria. Evidence clustered into two main streams. First, OSA was associated with frailty and sarcopenia-related phenotypes through mechanisms including intermittent hypoxia, systemic inflammation, sleep fragmentation, and neuroendocrine dysregulation. Second, in HNC, sarcopenia and frailty were associated with worse clinical outcomes, greater treatment burden, postoperative complications, treatment toxicity, and mortality. Direct evidence linking OSA with HNC within the included studies was limited. Therefore, the available evidence does not yet establish a fully demonstrated three-way clinical relationship among sarcopenia, frailty, and OSA in HNC.

**Conclusion:**

The available evidence supports clinically relevant overlap between OSA, frailty, and sarcopenia-related phenotypes, and a parallel association of sarcopenia and frailty with adverse HNC outcomes. However, the proposed triad should be interpreted as a hypothesis-generating framework rather than an established clinical entity. Future prospective studies should integrate objective OSA assessment, frailty evaluation, and radiological muscle measurements in HNC populations.

## Graphical Abstract

**Figure:**
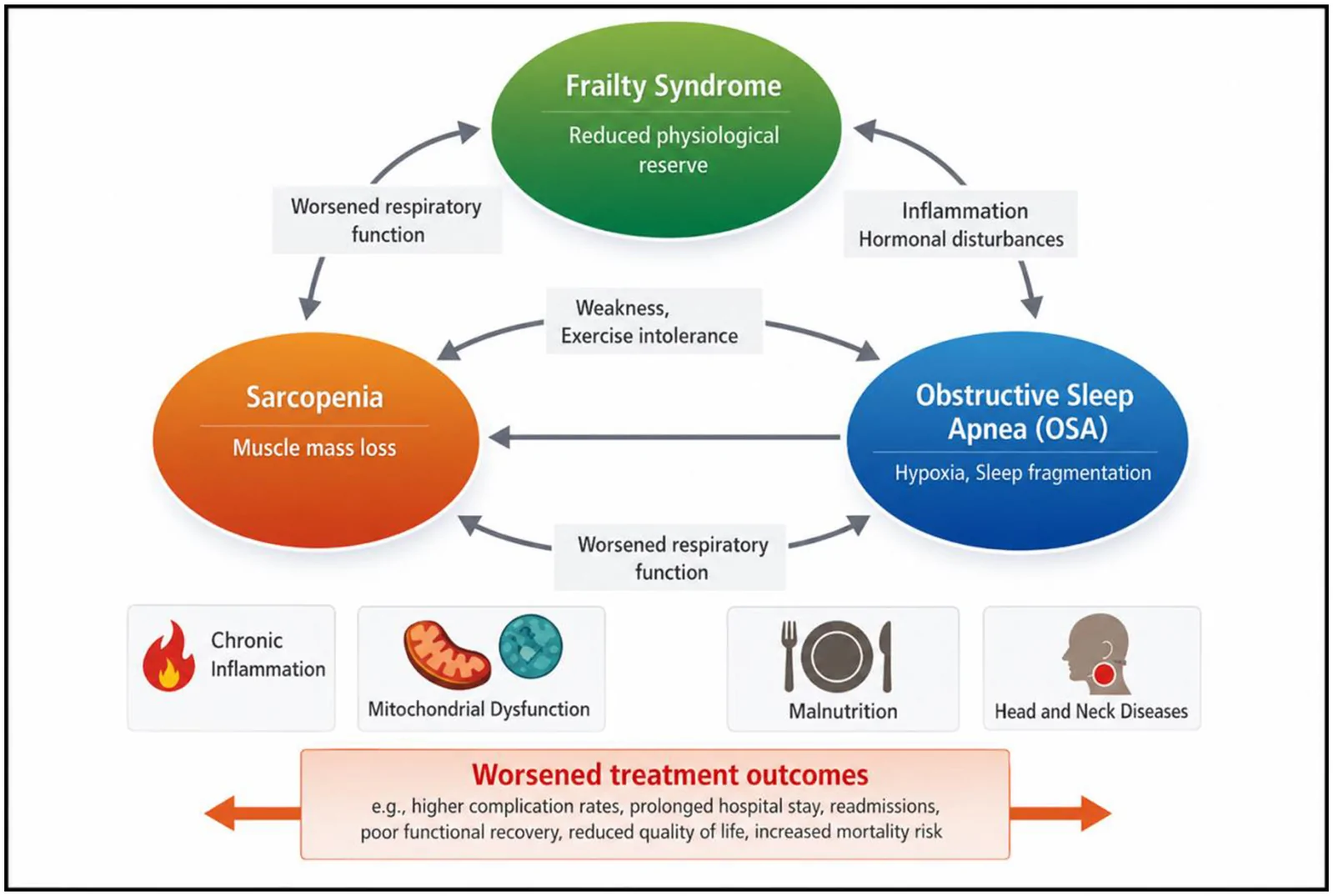
Conceptual pathophysiological model illustrating the proposed interplay between sarcopenia, frailty syndrome, and obstructive sleep apnea in head and neck cancer and upper airway disorders. The term adverse clinical outcomes refers to postoperative complications, treatment toxicity, reduced treatment tolerance, poorer survival, impaired functional recovery, and reduced physiological reserve. Created with Biorender.com.

## 1 Introduction

Population aging represents a major challenge for modern healthcare systems, contributing to an increasing burden of chronic diseases and geriatric syndromes. Among these, sarcopenia and frailty syndrome are key determinants of adverse clinical outcomes, including functional decline, hospitalization, postoperative complications, treatment intolerance, and mortality (1–4). Sarcopenia is characterized by a progressive loss of skeletal muscle mass and function, driven by complex mechanisms such as chronic low-grade inflammation, hormonal imbalance, mitochondrial dysfunction, and metabolic dysregulation. Frailty is a multidimensional state of increased vulnerability to stressors resulting from cumulative physiological decline (1, 2).

These two conditions frequently coexist and share overlapping biological pathways, including chronic inflammation, impaired anabolic signaling, and neuromuscular dysfunction. Their interaction reflects a broader aging phenotype characterized by reduced physiological reserve and reduced ability to recover after acute illness, surgery, oncologic treatment, or other stressors. In recent years, increasing attention has been directed toward sleep disorders, particularly obstructive sleep apnea, as potential contributors to accelerated biological aging and geriatric vulnerability (5, 6). In this review, the term aging phenotype refers to a clinically observable pattern of age-associated vulnerability, characterized by reduced physiological reserve, impaired functional resilience, increased susceptibility to stressors, and overlapping biological processes, including inflammation, altered body composition, sleep-related hypoxemia, and neuromuscular dysfunction. It is used as a conceptual framework rather than as a formally validated diagnostic category. OSA is defined by recurrent episodes of upper airway obstruction during sleep, leading to intermittent hypoxia, sleep fragmentation, and sympathetic nervous system activation. These disturbances are associated with systemic consequences including cardiovascular disease, metabolic dysfunction, cognitive impairment, and increased mortality (6, 7).

Emerging evidence suggests that OSA may be closely linked to both sarcopenia and frailty. Intermittent hypoxia and sleep disruption can impair hormonal regulation, including the growth hormone/IGF-1 axis, testosterone levels, and cortisol balance, thereby promoting muscle catabolism and functional decline (5). Additionally, systemic inflammation mediated by cytokines such as interleukin-6 and tumor necrosis factor-α may represent a shared biological pathway connecting these conditions (8). Epidemiological studies further indicate that OSA is associated with an increased prevalence of frailty, with disease severity correlating with higher vulnerability and worse outcomes in older adults (9, 10).

These interactions are particularly relevant in head and neck cancer and upper airway disorders, where structural and functional impairments may affect breathing, swallowing, nutritional status, airway stability, and treatment tolerance. While OSA represents a prototypical disorder within this group, increasing attention has also been given to the concept of “respiratory sarcopenia,” highlighting the role of muscle dysfunction in airway stability and ventilatory efficiency (11, 12). A further clinically relevant issue is the relationship between OSA and HNC. Although direct evidence combining OSA, sarcopenia, and frailty in the same HNC cohorts remains scarce, recent literature suggests that OSA and HNC may be bidirectionally related. OSA may influence tumor biology and patient-reported outcomes through intermittent hypoxia and systemic effects, whereas HNC and its treatment, particularly radiotherapy and surgery affecting upper airway anatomy, may contribute to the development or persistence of OSA (13). This evidence provides a rationale for considering OSA when interpreting geriatric vulnerability in upper respiratory tract oncology, while also emphasizing that the integrated model remains hypothesis-generating.

Despite a growing body of literature, existing evidence remains heterogeneous, and the relationships between sarcopenia, frailty, and OSA have not been comprehensively synthesized in the context of upper respiratory tract diseases. There is a lack of integrative analyses combining mechanistic insights, clinical associations, and potential therapeutic implications.

Therefore, the aim of this scoping review was to systematically map the current evidence on sarcopenia, frailty syndrome, and OSA in head and neck cancer and upper airway disorders, with particular attention to whether the available literature supports an integrated clinical model linking OSA-related vulnerability with sarcopenia and frailty in HNC and related disorders. To preserve narrative precision, this review does not treat, HNC, OSA, sarcopenia, frailty, and aging as equivalent constructs. Instead, these conditions are considered clinically related but distinct domains that may converge in selected older patients with upper airway disease or head and neck cancer.

## 2 Materials and methods

### 2.1 Study design

This scoping review was conducted to systematically map and synthesize the available evidence regarding the associations between sarcopenia, frailty, and obstructive sleep apnea as components of the aging phenotype in upper respiratory tract diseases.

The methodology was based on the Joanna Briggs Institute (JBI) framework for scoping reviews and reported according to the Preferred Reporting Items for Systematic Reviews and Meta-Analyses for Scoping Reviews (PRISMA-ScR) extension. A completed PRISMA ScR checklist is provided as Supplementary Table S1.

A scoping review approach was chosen because of the heterogeneity, conceptual complexity, and emerging nature of the evidence base, which precluded a meaningful quantitative synthesis (14–17).

### 2.2 Eligibility criteria

Eligibility criteria were established using the Population–Concept–Context (PCC) model recommended for scoping reviews (15–17).

#### 2.2.1 Population

Studies involving adult patients were considered eligible, with a particular focus on older and geriatric populations. The review focused on patients with upper respiratory tract diseases, including, but not limited to, obstructive sleep apnea, head and neck cancer, and functional upper respiratory disorders affecting breathing and swallowing.

#### 2.2.2 Concept

The concept of interest included sarcopenia, frailty, obstructive sleep apnea, and the biological and clinical relationships between these conditions as components of the aging phenotype. Studies were eligible if they addressed at least one of these areas or their interactions in the context of upper respiratory tract diseases.

#### 2.2.3 Context

The context included clinical, epidemiological, translational, and review evidence related to upper respiratory tract diseases. No restrictions were placed on healthcare settings.

### 2.3 Inclusion and exclusion criteria

#### 2.3.1 Inclusion criteria

- studies published between January 1, 2015 and December 31, 2025;
- full-text articles available;
- publications in English;
- adult populations;
- studies addressing sarcopenia, frailty, OSA, or their interrelationships;
- relevance to upper respiratory tract diseases;
- original research (prospective, retrospective, cross-sectional, case-control, cohort), interventional studies, population-based analyses, or review articles providing clinically or mechanistically relevant evidence.

#### 2.3.2 Exclusion criteria

- pediatric populations;
- lack of relevance to upper respiratory tract diseases;
- absence of sarcopenia, frailty, or OSA within the study scope;
- conference abstracts without full text;
- editorials, commentaries, letters, and expert opinions without analytical contribution;
- duplicate records;
- studies outside the defined time frame.

During full-text screening, studies were additionally excluded if:

- they lacked sufficient methodological or conceptual relevance;
- they did not provide extractable data related to the relationships of interest;
- they focused primarily on unrelated organ systems;
- they contributed only marginal or redundant information.

### 2.4 Information sources and search strategy

A comprehensive literature search of PubMed, EBSCO, and Google Scholar was conducted to identify potentially relevant studies published between January 1, 2015, and December 31, 2025. PubMed was used as the primary biomedical database, with EBSCO and Google Scholar as supplementary sources to enhance sensitivity to clinically heterogeneous literature.

The search strategy was developed using a combination of controlled vocabulary and free-text terms related to sarcopenia, frailty syndrome, obstructive sleep apnea, and upper respiratory tract diseases. The primary search strategy in PubMed was as follows: “sarcopenia,” “frailty syndrome,” “frailty index,” “obstructive sleep apnea,” “OSA,” “head and neck cancer,” “upper respiratory tract,” “swallowing,” and “otolaryngology.” Boolean operators (AND, OR) were used to refine the search strategy.

Equivalent keyword combinations adapted to the indexing system and search interface of each database were applied to EBSCO and Google Scholar. The bibliographic lists of eligible articles were also manually searched to identify additional relevant records.

The final search was conducted on December 31, 2025. All records were exported to a bibliography management file, and duplicates were removed before screening. The search and selection workflow was documented in accordance with PRISMA-ScR recommendations (15–17).

The study selection process was conducted in two stages (title/abstract screening and full-text assessment). Discrepancies were resolved through consensus among the authors.

### 2.5 Study selection

All identified records were exported to a reference management tool, and duplicates were removed prior to screening.

The study selection process was conducted in two stages:

- title and abstract screening;
- full-text assessment.

Screening was performed independently by two reviewers. Discrepancies were resolved through discussion and consensus.

**Figure 1:**
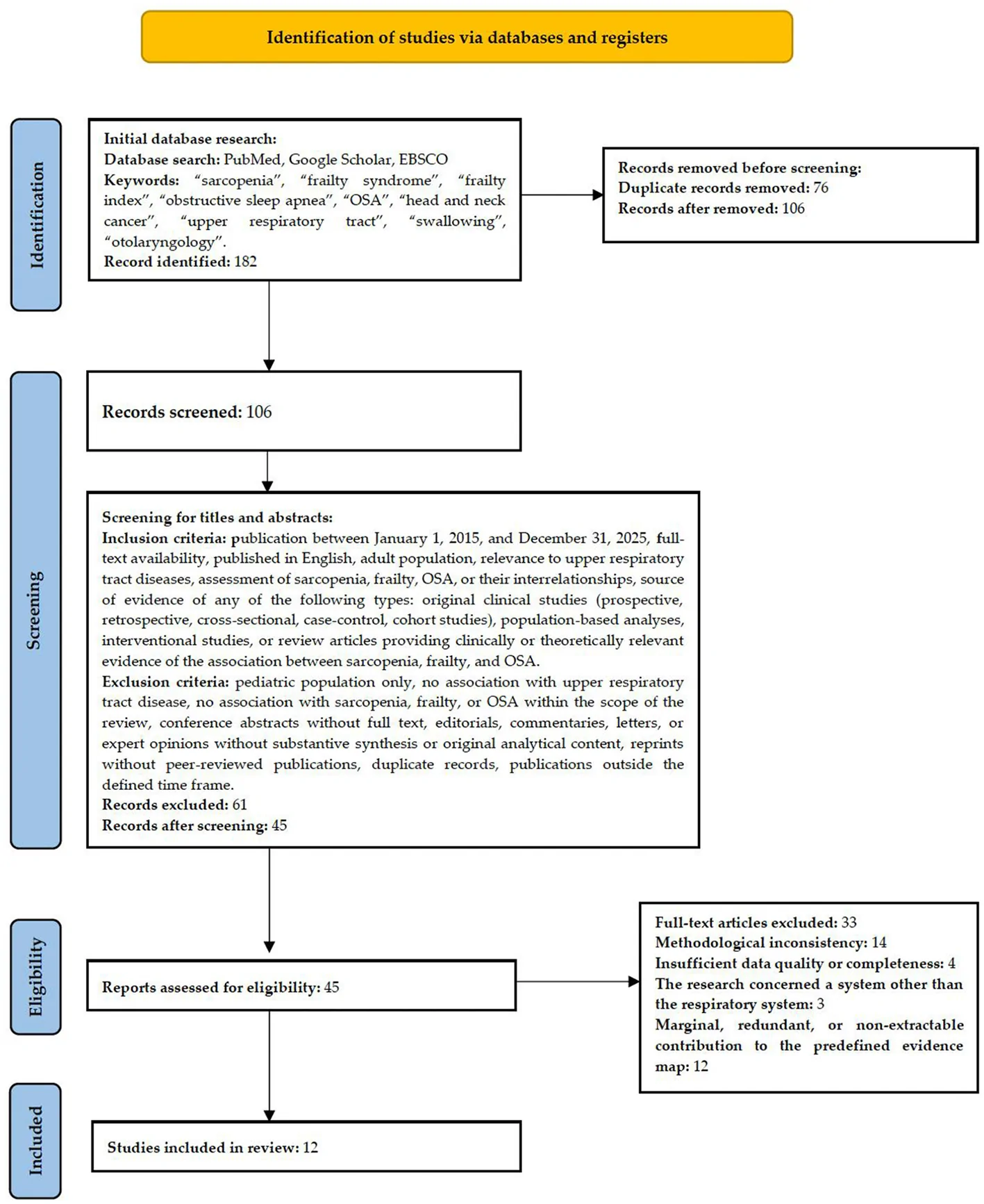
PRISMA ScR flow diagram showing identification, duplicate removal, title and abstract screening, full text eligibility assessment, reasons for full text exclusion, and final inclusion of studies.

The study selection process is presented in a PRISMA flow diagram (Figure 1). The database search identified 182 records. After removing 76 duplicate records, 106 records were screened by title and abstract. A total of 61 records were excluded at this stage. Forty-five reports were assessed for eligibility in full text. Thirty-three full-text articles were excluded for the following reasons: methodological inconsistency, *n* = 14; insufficient data quality or completeness, *n* = 4; focus on a system other than the respiratory system, *n* = 3; marginal, redundant, or non-extractable contribution to the pre-defined evidence map, *n* = 12. Twelve studies were included in the final qualitative synthesis.

### 2.6 Data charting (data extraction)

Data extraction (charting) was performed in accordance with JBI recommendations.

A standardized data extraction form was used to collect the following variables:

- first author and year of publication;
- clinical area;
- study design;
- population characteristics;
- assessed variables (sarcopenia, frailty, OSA);
- key findings.

Data were extracted independently by two reviewers and cross-checked for consistency. The extracted data were organized using Microsoft Excel.

### 2.7 Critical appraisal of individual sources

In line with the JBI methodology for scoping reviews, a formal risk-of-bias assessment was not performed because the primary objective was to map the available evidence rather than to generate pooled effect estimates (15–17).

However, during full-text screening, studies with substantial methodological limitations, insufficient reporting quality, or limited relevance to the PCC framework were excluded. Additionally, key methodological characteristics of the included studies were described to support the interpretation of the findings (16, 17).

### 2.8 Data synthesis

Given the heterogeneity of study designs, populations, diagnostic approaches, and outcome measures, quantitative synthesis was not feasible. Results were synthesized qualitatively using a thematic mapping approach. Extracted data were first organized according to clinical context, study design, population, assessment method, and reported association. Studies were then grouped into three pre-defined domains: associations between OSA and frailty, associations between OSA and sarcopenia, and the role of sarcopenia and frailty in HNC.

Within each domain, findings were compared according to whether they primarily addressed mechanistic plausibility, clinical association, prognostic value, or treatment-related outcomes. This structure allowed the evidence to be mapped without assuming causality or a direct three-way relationship unless such a relationship was explicitly examined in the included studies. The synthesis therefore distinguishes between evidence directly observed in the included studies and broader contextual evidence used to interpret potential links between OSA and HNC.

Because some included sources were narrative or review-based, these records were not interpreted as primary evidence for associations, prognostic effects, or treatment-related outcomes. They were used to map concepts, summarize existing clinical perspectives, and identify gaps in the evidence base. Conclusions regarding clinical associations were based primarily on original observational, population-based, interventional, or cohort studies, while review-based sources were treated as supportive contextual evidence.

## 3 Results

### 3.1 Characteristics of included studies

A total of 12 studies met the eligibility criteria and were included in the final qualitative synthesis. The included literature was methodologically heterogeneous and consisted pre-dominantly of observational studies, with only limited interventional evidence. The studies covered three main thematic areas: associations between obstructive sleep apnea and frailty, associations between OSA and sarcopenia, and the role of sarcopenia and frailty in head and neck cancer (3, 4, 7, 18–24). Importantly, no included study directly evaluated OSA, sarcopenia, and frailty simultaneously within the same HNC cohort. The evidence maps therefore consisted of two related but distinct streams: OSA-associated geriatric vulnerability and sarcopenia- or frailty-associated vulnerability in HNC. This distinction was maintained throughout the synthesis to avoid overinterpreting indirect evidence.

Regarding study design, the evidence base included prospective observational studies, retrospective analyses, cross-sectional studies, population-based analyses, a propensity score matching study, and narrative or non-systematic reviews (7, 18–23, 25–28). Most studies focused either on older adults with OSA or on patients with head and neck malignancies, particularly elderly patients with HNC and HNSCC (7, 18, 19, 22–28). Review-based articles were retained only when they provided clinically relevant synthesis for the evidence map. They were not treated as independent patient-level evidence for clinical associations, prognostic effects, or treatment outcomes.

The HNC-related literature has mainly evaluated frailty status, sarcopenia, and the skeletal muscle index (SMI), and their associations with treatment tolerance and clinical outcomes (18–22, 24, 26). In contrast, studies conducted in OSA populations primarily assessed frailty risk, sarcopenia risk, sarcopenic obesity, and the potential association between OSA severity or treatment and age-related vulnerability (7, 23, 27–29) (Table 1).

**Table 1:** Characteristics of studies included in the scoping review evaluating the relationships between sarcopenia, frailty syndrome, and obstructive sleep apnea as components of the aging phenotype in upper respiratory tract diseases.

Author, year	Clinical area	Study type	Population	OSA assessment or case definition	What was assessed	Key finding	References
Kwon et al., 2016	Head and neck cancers (HNC)	Prospective observational study	Elderly patients with HNC, *n* = 165	Not applicable	HNC- specific frailty index with emphasis on respiratory and swallowing functions	Functional respiratory and swallowing deficits were significantly associated with early morbidity and mortality	(18)
Nieman et al., 2018	HNC surgery	Observational study	Adults from the nationwide inpatient sample undergoing ablative surgery for malignant oral cavity, laryngeal, hypopharyngeal, or oropharyngeal neoplasm, 2001 to 2010, *n* = 159, 301	Not applicable	Frailty defined using the Johns Hopkins adjusted clinical groups frailty indicator; in hospital mortality, postoperative complications, length of stay, and hospitalization costs	Frailty independently predicted postoperative morbidity, mortality, length of stay, and hospitalization costs in patients undergoing HNC surgery.	(19)
Noor et al., 2018	Geriatric head and neck oncology	Narrative review	Literature review	Not applicable	State of knowledge on frailty in HNC	It confirms the growing role of frailty assessment in geriatric patients with HNC and justifies the inclusion of frailty in therapeutic decisions	(20)
de Bree et al., 2022	HNC	Review	Literature review	Not applicable	Measurement of sarcopenia in HNC and association with frailty	Sarcopenia in HNC may serve as a practical marker of risk for adverse outcomes and is associated with a frailty phenotype	(21)
Meerkerk et al., 2022	Elderly patients with HNC	Observational study	Patients aged 70 years or older with HNC, *n* = 73	Not applicable	Skeletal muscle mass measured on routine diagnostic imaging, SMI, sarcopenia, CGA defined frailty, GFI, and Fried criteria	Low SMI and sarcopenia were associated with CGA defined frailty. Low SMI may support rapid frailty screening in older patients with HNC.	(22)
Mehawej et al., 2022	OSA in older adults	Cross-sectional study/multivariate analysis	Older adults with atrial fibrillation, *n* = 970	STOP BANG based OSA risk assessment, not PSG confirmed diagnosis	OSA risk and frailty, cognitive function and quality of life	The risk of OSA correlated with geriatric disorders, including frailty	(25)
Zwart et al., 2023	Skin cancer of the head and neck	Retrospective study on prospectively collected data	Patients surgically treated for cutaneous malignancies of the head and neck with available neck imaging and geriatric assessment, *n* = 57	Not applicable	C3 derived SMI from CT or MRI, radiologically defined sarcopenia, frailty screening, malnutrition risk, and postoperative complications	Radiologically defined sarcopenia was associated with frailty and malnutrition risk and may serve as a practical biomarker of vulnerability in patients with head and neck skin cáncer	(26)
Kim et al., 2023	OSA	Clinical trial	Patients with PSG confirmed OSA, *n* = 40, and healthy controls, *n* = 52	PSG confirmed OSA; temporal muscle thickness assessed on brain MRI	Temporal muscle thickness on brain MRI, AHI, lowest oxygen saturation, and clinical variables related to OSA severity	Temporal muscle thickness did not differ significantly between OSA patients and controls, but it was negatively correlated with the lowest oxygen saturation in the OSA group, supporting a potential link between nocturnal hypoxemia and muscle related vulnerability	(27)
Tao et al., 2024	OSA	Population-based cross-sectional study	Adults included in the NHANES 2015 to 2018 population-based analysis	OSA status defined according to the original NHANES based study definition; not a PSG based clinical cohort	OSA status, early onset sarcopenia, sarcopenic obesity, and body composition related variables	OSA was associated with an increased risk of early onset sarcopenia and sarcopenic obesity	(28)
Xue et al., 2024	OSA in older men	Multicenter study	Elderly male OSAS; number of respondents not reported in available metadata	Clinically diagnosed OSAS in elderly male patients	Risk of sarcopenia and osteoporosis in OSAS	Strengthens the idea that OSA in older patients is correlated with the musculoskeletal component of the aging phenotype	(23)
Xue et al., 2024	OSA in older people	Propensity scores matching analysis	Elderly OSA; number of respondents not reported in metadata	OSA diagnosis with CPAP exposure compared with no CPAP after propensity score matching	Impact of CPAP on incident frailty	CPAP was associated with a lower risk of developing frailty, which has significant translational significance and strengthens the OSA–frailty association	(7)
Giannotti et al., 2024	HNC radiotherapy	Prospective analysis	Patients aged 65 years or older with newly diagnosed HNSCC undergoing radiotherapy with or without chemotherapy, *n* = 117	Not applicable	Pretreatment frailty assessed using a 40 item frailty index, overall survival, and acute radiotherapy toxicity	Pretreatment frailty assessment was associated with overall survival in older patients with HNSCC undergoing radiotherapy and may improve pre-treatment risk stratification.	(24)

OSA, obstructive sleep apnea; OSAS, obstructive sleep apnea syndrome; HNC, head and neck cancer; HNSCC, head and neck squamous cell carcinoma; SMI, skeletal muscle index; C3, third cervical vertebra; CT, computed tomography; MRI, magnetic resonance imaging; PSG, polysomnography; AHI, apnea hypopnea index; CPAP, continuous positive airway pressure; STOP BANG, snoring, tiredness, observed apnea, high blood pressure, body mass index, age, neck circumference, gender screening questionnaire for OSA risk; NHANES, national health and nutrition examination survey; CGA, comprehensive geriatric assessment; GFI, Groningen frailty indicator; RT, radiotherapy; n, number of participants; Ref., reference.

Narrative and review-based articles were included to map the conceptual and clinical context. They were not interpreted as primary evidence for associations, prognostic effects, treatment effects, or patient-level outcomes.

### 3.2 Association between OSA and frailty

Evidence from the included studies consistently indicated an association between OSA and frailty in older populations. In a cross-sectional, multivariate analysis of older adults with atrial fibrillation, Mehawej et al. (25) demonstrated that increased OSA risk, assessed with the STOP-BANG questionnaire, was associated with geriatric syndromes, including frailty, as well as poorer cognitive and quality-of-life measures.

Additional support for the association between OSA and frailty was provided by studies in elderly OSA populations. Xue et al. (7) reported that, among older patients with OSA, CPAP treatment was associated with a lower risk of incident frailty in a propensity score-matched analysis, suggesting that frailty-related outcomes may differ by treatment exposure. In another multicenter cohort, the same group examined oxygenation-related markers, particularly mean nocturnal pulse oxygen saturation (MSpO_2_) and time spent with oxygen saturation below 90 percent. Abnormal nocturnal oxygenation was associated with incident frailty, supporting the clinical relevance of nocturnal oxygenation as a frailty-related physiological marker in older adults with OSA (10).

Taken together, the included evidence indicates that frailty is a recurrent and clinically relevant finding in older adults with OSA, and that this relationship has been observed across both observational and treatment-based analyses (7, 10, 25).

### 3.3 Association between OSA and sarcopenia

The included studies also showed a consistent relationship between OSA and sarcopenia-related phenotypes. In a population-based cross-sectional analysis of NHANES 2015–2018 data, Tao et al. (28) found that OSA was associated with an increased risk of early sarcopenia and sarcopenic obesity. This finding extended the OSA-related phenotype beyond sleep disturbance alone and linked it to adverse changes in body composition.

Similarly, Xue et al. (23) demonstrated in elderly men with OSA that obstructive sleep apnea syndrome was associated with a higher risk of sarcopenia and osteoporosis, reinforcing the relationship between OSA and musculoskeletal vulnerability in older patients. In addition, Kim et al. evaluated 40 patients with PSG-confirmed OSA and 52 healthy controls, using temporal muscle thickness (TMT) on brain MRI as an imaging marker of sarcopenia. Although TMT did not differ significantly between OSA patients and controls, TMT was negatively correlated with the lowest oxygen saturation in the OSA group. These findings do not prove that OSA causes sarcopenia, but they support a biologically plausible association between nocturnal hypoxemia and reduced muscle-related reserve in OSA (27).

Across the included studies, the OSA–sarcopenia association was observed in both population-level and clinical settings, with recurrent associations with reduced muscle-related reserve and unfavorable body composition profiles (23, 27, 28).

### 3.4 Sarcopenia and frailty in head and neck cancer

The largest clinically coherent subgroup of included studies concerned patients with head and neck cancer. Across this body of evidence, both frailty and sarcopenia were repeatedly associated with poorer outcomes, higher treatment burden, and reduced physiological reserve (18–22, 24, 26).

Frailty was shown to be clinically meaningful in both surgical and non-surgical HNC settings. Kwon et al., (18) in a prospective observational study of elderly patients with HNC (*n* = 165), introduced a head and neck cancer-specific frailty index with particular emphasis on respiratory and swallowing functions. Functional deficits in these domains were significantly associated with early morbidity and mortality. In patients undergoing head and neck cancer surgery, Nieman et al. (19) reported that frailty worsened short-term postoperative outcomes. In addition, Noor et al. (20) summarized the growing relevance of frailty assessment in geriatric head and neck oncology and emphasized its potential role in treatment decision-making.

Sarcopenia-related findings were likewise consistent. de Bree et al. (21) reported that sarcopenia in HNC may serve as a practical marker of risk for adverse outcomes and is associated with a frailty phenotype. Meerkerk et al. (22) showed that low skeletal muscle mass and sarcopenia were associated with frailty in elderly patients with HNC and suggested that low SMI may serve as a rapidly available imaging-based marker to support frailty screening in older HNC patients. Similarly, Zwart et al. (26) demonstrated that radiologically defined sarcopenia may function as a practical biomarker of frailty and malnutrition in patients with head and neck skin cancer.

The prognostic significance of frailty in oncologic treatment was further supported by Giannotti et al., (24) who found that, in older patients undergoing radiotherapy for HNSCC, pre-treatment frailty assessment helped predict both survival and treatment-related toxicity. Overall, the HNC-related evidence consistently showed that frailty and sarcopenia are not isolated descriptive findings, but clinically relevant markers associated with worse outcomes and potentially useful for risk stratification (18–22, 24, 26).

### 3.5 Summary of the evidence map

Across the 12 included studies, two dominant evidence streams emerged. First, in older adults with OSA, frailty and sarcopenia-related phenotypes were repeatedly associated with sleep-disordered breathing, nocturnal oxygenation, or CPAP exposure (7, 10, 23, 25, 27, 28). Second, in head and neck oncology, frailty and sarcopenia were consistently associated with worse prognosis, greater treatment burden, and adverse clinical outcomes, while radiological assessment of muscle mass appeared to offer a potentially practical approach for identifying vulnerable patients (14 to 18, 20, 24). Importantly, the included studies did not directly examine the full three-way relationship among OSA, sarcopenia, and frailty within the same HNC cohorts. Therefore, the evidence map supports conceptual convergence and research prioritization rather than confirmation of a single established clinical entity.

## 4 Discussion

The present scoping review maps evidence on sarcopenia, frailty syndrome, and OSA in upper respiratory tract diseases. The available literature supports two clinically important evidence streams: associations between OSA and geriatric vulnerability, expressed as frailty- and sarcopenia-related phenotypes, and associations between sarcopenia or frailty and adverse outcomes in patients with HNC. However, the included studies do not yet establish a proven three-way clinical entity linking sarcopenia, frailty, and OSA within the same HNC populations. Therefore, the integrated model proposed in this review should be interpreted as a hypothesis-generating framework rather than as a confirmed triad.

### 4.1 OSA as a potentially modifiable component associated with the aging phenotype

One of the key observations emerging from this review is the consistent association between OSA and both frailty and sarcopenia-related phenotypes. Across multiple studies, OSA was associated with a higher prevalence of frailty in older adults, as demonstrated in both cross-sectional and population-based analyses (7, 10, 25, 30). Furthermore, evidence suggests that CPAP therapy may reduce the incidence of frailty, supporting the hypothesis that OSA is a modifiable component of age-related vulnerability (7).

Similarly, associations between OSA and sarcopenia were observed across diverse study designs, including population-based analyses and clinical cohorts (23, 27, 28). These findings extend the clinical significance of OSA beyond sleep-related symptoms, suggesting its involvement in systemic processes that affect muscle mass, body composition, and functional capacity.

Taken together, these results indicate that OSA should be interpreted not only as a localized disorder of upper airway obstruction, but also as a condition with systemic implications that may contribute to accelerated biological aging.

### 4.2 Shared pathophysiological mechanisms

The observed relationships between OSA, sarcopenia, and frailty can be explained by several overlapping biological mechanisms. Intermittent hypoxia, a hallmark of OSA, activates inflammatory pathways and increases the production of pro-inflammatory cytokines, such as interleukin-6 and tumor necrosis factor-α, which promote muscle catabolism and functional decline. In parallel, oxidative stress and mitochondrial dysfunction contribute to impaired muscle metabolism and reduced physical performance (8, 31).

Neuroendocrine dysregulation represents another important pathway linking these conditions. OSA-related disturbances in the growth hormone/IGF-1 axis, testosterone levels, and cortisol secretion may shift the balance toward catabolic processes, thereby promoting the development of sarcopenia and contributing to frailty. Additionally, sleep fragmentation and reduced sleep quality may further exacerbate metabolic and hormonal disturbances (5, 6).

The concept of respiratory sarcopenia may provide a useful framework for considering the relationship between skeletal muscle dysfunction and respiratory impairment. However, it should be interpreted with caution because the term is still evolving and not uniformly defined in the literature. Some authors use it to describe reduced respiratory muscle strength, whereas others emphasize global sarcopenia with respiratory consequences or impaired ventilatory reserve. In the context of OSA and HNC, the concept is therefore best regarded as a mechanistic hypothesis rather than a validated clinical construct. Reduced pharyngeal or respiratory muscle function may plausibly affect airway stability and ventilatory efficiency, while OSA-related hypoxemia and inflammation may coexist with muscle-related vulnerability. Nevertheless, the included studies do not directly establish respiratory sarcopenia as a causal mediator linking OSA, frailty, and HNC outcomes (29, 32, 33).

### 4.3 Clinical relevance in head and neck cancer

The clinical relevance of these overlapping domains is particularly important in patients with head and neck cancer, where structural disease, functional impairment, dysphagia, malnutrition, impaired airway function, and reduced physiological reserve may coexist. The included studies consistently demonstrated that both sarcopenia and frailty are associated with poorer clinical outcomes, including increased treatment toxicity, reduced survival, and higher complication rates (18–22, 26, 34, 35).

Frailty assessment appears to provide important prognostic information beyond traditional clinical parameters such as chronological age or performance status. Studies in surgical and non-surgical HNC populations indicate that frailty is associated with worse short-term and long-term outcomes, supporting its role as an independent marker of biological vulnerability (18–20, 24, 36).

At the same time, sarcopenia—particularly when assessed using radiological methods—emerges as a practical and objective biomarker. Imaging-based measures, such as the SMI, enable identification of patients with reduced physiological reserve without additional diagnostic burden (21, 22, 26, 37). The convergence of these findings suggests that sarcopenia and frailty should be considered complementary tools in risk stratification. This interpretation is further supported by Bergamaschi et al., who evaluated sarcopenia in patients with oropharyngeal cancer undergoing radiotherapy and showed that sarcopenia occurring during treatment may have prognostic relevance. Their findings reinforce the clinical importance of monitoring muscle loss during radiotherapy and support early nutritional and supportive interventions in patients with head and neck cancer (38).

Importantly, HNC represents a unique clinical context in which disease-related factors such as dysphagia, malnutrition, and impaired airway function directly contribute to the progression of sarcopenia and frailty. This interaction may accelerate the aging phenotype and further worsen treatment tolerance and outcomes (39).

### 4.4 OSA and HNC as a contextual bridge

The reviewer's concern is important because the included studies primarily examined either OSA in relation to frailty or sarcopenia, or sarcopenia and frailty in HNC. Direct studies evaluating OSA, sarcopenia, and frailty together in HNC populations were not identified among the included records. This limits the strength of any conclusion claiming a fully established triad.

Nevertheless, evidence outside the included evidence map suggests that OSA and HNC may be clinically connected. A recent systematic review reported a bidirectional relationship in which OSA may influence HNC biology and outcomes, while HNC treatment, especially radiotherapy and anatomical changes affecting the upper airway, may contribute to OSA development or persistence (13). This literature provides a plausible bridge between the OSA- and HNC-focused evidence streams, but it should be used with caution as contextual support rather than as proof that sarcopenia, frailty, and OSA constitute a single established clinical syndrome in HNC.

Accordingly, the clinical value of the present review lies in identifying converging domains of vulnerability that may coexist in older patients with upper respiratory tract disease. The strongest current evidence supports integrated screening and prospective research, not definitive causal integration.

### 4.5 Integrated model of the aging phenotype

Based on the available evidence, sarcopenia, frailty, and OSA may be conceptualized as partially overlapping markers of an aging phenotype characterized by reduced physiological reserve, impaired resilience, and increased susceptibility to stressors. This model remains conceptual and should not be interpreted as proof of a single established clinical trial, particularly in HNC populations.

In this model, OSA acts as a systemic stressor through mechanisms such as intermittent hypoxia and neuroendocrine dysregulation, contributing to muscle loss and functional decline. Sarcopenia reflects the structural and functional consequences of these processes, while frailty represents their cumulative clinical expression.

Emerging data, including Mendelian randomization analyses, suggest that the relationship between sarcopenia-related traits and OSA may have a causal component, further strengthening the biological plausibility of this integrated model (8). Additionally, body composition and fat distribution appear to influence both muscle function and airway mechanics, linking metabolic and respiratory aspects of the aging phenotype (3, 4).

From a clinical perspective, the coexistence of these conditions may define a subgroup of patients with particularly high vulnerability, characterized by increased risk of complications, poorer treatment tolerance, and reduced capacity for recovery.

## 5 Clinical implications

The findings of this review have several clinical implications.

First, in patients with HNC, assessment of frailty and sarcopenia should be considered as part of pre-treatment risk stratification, because both conditions are associated with postoperative complications, treatment burden, reduced survival, and other adverse outcomes. Imaging-based assessment of skeletal muscle mass may be particularly useful in HNC because CT or MRI is frequently obtained as part of the routine oncologic workup.

Second, in older patients with OSA, evaluation of frailty and muscle-related vulnerability may help identify individuals at increased risk of functional decline. Available evidence also suggests that CPAP exposure may be associated with a lower incidence of frailty, although causality cannot be established from the available observational data.

Third, in older HNC patients, especially those with symptoms of sleep-disordered breathing or with anatomical or treatment-related airway changes, OSA should be considered as a potentially relevant comorbidity. At present, this recommendation is based on contextual OSA and HNC literature and biological plausibility rather than on direct studies integrating OSA, sarcopenia, and frailty in the same HNC cohorts.

## 6 Limitations of the evidence

This scoping review has several significant limitations.

First, the number of included studies was limited, and the studies were methodologically heterogeneous. Second, most of the included studies were observational, which limits causal inference. Third, the evidence did not directly test the full relationship among OSA, sarcopenia, and frailty within HNC populations. Therefore, the proposed integrated model should be regarded as hypothesis-generating. Fourth, definitions and assessment methods for sarcopenia, frailty, and OSA differed across studies. OSA was assessed using different approaches, including screening questionnaires, PSG-confirmed diagnosis, and population-based survey variables. Fifth, the literature search was limited to selected databases and English-language publications, which may have led to the omission of relevant studies. Finally, although recent OSA and HNC literature supports a contextual bridge, those data should be clearly distinguished from the studies included in the formal evidence map unless the search strategy is updated and the PRISMA flow diagram is revised accordingly.

In accordance with scoping review methodology, the aim of this study was to map the available evidence and identify research gaps rather than to estimate pooled effects or establish causality.

## 7 Future directions

Future research should focus on prospective cohorts that assess OSA, sarcopenia, and frailty simultaneously in patients with HNC and other upper respiratory tract diseases. Such studies should use objective OSA assessment, preferably PSG or validated home sleep apnea testing, standardized frailty tools, and reproducible muscle measurements from routinely acquired CT or MRI.

Interventional studies are also needed to determine whether targeted management can modify outcomes. Relevant interventions include CPAP or other OSA treatments, nutritional support, resistance exercise, pre-habilitation, dysphagia management, and integrated geriatric oncology pathways. In HNC, future studies should evaluate whether combining OSA status, frailty assessment, and imaging-based sarcopenia markers improves prediction of treatment toxicity, postoperative complications, survival, functional recovery, and quality of life.

## 8 Conclusions

The available evidence supports clinically relevant overlap between OSA and geriatric vulnerability, expressed through frailty and sarcopenia-related phenotypes. Separately, sarcopenia and frailty are consistently associated with adverse outcomes in patients with HNC. However, the included studies do not demonstrate that sarcopenia, frailty, and OSA constitute a single established clinical entity in head and neck cancer or upper airway disorders.

A more accurate interpretation is that these conditions represent converging markers of reduced physiological reserve, with a plausible but insufficiently tested connection in HNC. Integrated assessment may improve risk stratification and support personalized management, but prospective studies are needed to determine whether OSA, sarcopenia, and frailty interact directly and whether targeted interventions improve clinical outcomes.

## Data Availability

The original contributions presented in the study are included in the article/supplementary material, further inquiries can be directed to the corresponding author.
