# Supplementary material for: Sarcopenia, frailty syndrome and obstructive sleep apnea as elements of the aging phenotype in head and neck cancer and upper airway disorders: a scoping review
Source: Front Med (Lausanne). 2026 Jul 30;13:1858903. doi: 10.3389/fmed.2026.1858903 (PMC13508823; doi:10.3389/fmed.2026.1858903)
Supplement: Supplementary file 1 [file Table_1.docx]

**Supplementary Table S1. PRISMA-ScR checklist**

*Manuscript: Sarcopenia, frailty syndrome and obstructive sleep apnea as elements of the aging phenotype in upper respiratory tract diseases: a scoping review*

This supplementary checklist follows the PRISMA-ScR reporting structure for scoping reviews. Section references refer to the revised manuscript submitted with the response to reviewers. Items 12 and 16 are optional PRISMA-ScR items and are marked as not applicable when no formal critical appraisal was performed.

| **Section** | **Item** | **Topic** | **PRISMA-ScR reporting item** | **Location in manuscript** | **Reporting status and comments** |
| --- | --- | --- | --- | --- | --- |
| Title | 1 | Title identification | Identify the article as a scoping review. | Title page | Reported. The title explicitly identifies the manuscript as a scoping review. |
| Abstract | 2 | Structured summary | Provide a structured summary including background, objectives, eligibility criteria, sources of evidence, charting methods, results, and conclusions as applicable. | Abstract | Reported. The abstract includes background, objective, methods, results, and conclusion. |
| Introduction | 3 | Rationale | Describe the rationale for the review and explain why a scoping review approach is appropriate in relation to what is already known. | Introduction | Reported. The introduction describes population aging, sarcopenia, frailty, OSA, HNC, respiratory sarcopenia, and the evidence gap. |
| Introduction | 4 | Objectives | State the review questions and objectives, ideally using key elements such as population, concept, and context. | End of Introduction | Reported. The objective is to map evidence on sarcopenia, frailty syndrome, and OSA in upper respiratory tract diseases, with attention to OSA related vulnerability and HNC. |
| Methods | 5 | Protocol and registration | Indicate whether a protocol exists, where it can be accessed, and whether registration information is available. | Section 2.1 Study Design | No separate public protocol registration is reported. The manuscript states that the review was conducted according to the JBI framework and reported according to PRISMA-ScR. |
| Methods | 6 | Eligibility criteria | Specify the characteristics of eligible sources of evidence and provide the rationale for inclusion and exclusion decisions. | Sections 2.2 and 2.3 | Reported. The manuscript defines the PCC framework, inclusion criteria, exclusion criteria, and additional full text exclusion criteria. |
| Methods | 7 | Information sources | Describe all information sources and indicate the date of the last search. | Section 2.4 Information sources and search strategy | Reported. PubMed, EBSCO, Google Scholar, and manual reference list searching are described. The final search date is reported as December 31, 2025. |
| Methods | 8 | Search strategy | Present the search strategy for at least one database with enough detail to allow repetition, including limits used. | Section 2.4 Information sources and search strategy | Reported in summary form. The manuscript lists the main search terms, databases, date limits, language restriction, and use of Boolean operators. |
| Methods | 9 | Selection of sources of evidence | State the process used to select sources of evidence for inclusion. | Sections 2.4 and 2.5 | Reported. Two stage screening, title and abstract screening, full text assessment, independent screening by two reviewers, and consensus resolution are described. |
| Methods | 10 | Data charting process | Describe the method used to chart data from included sources and any process for checking or confirming the extracted data. | Section 2.6 Data charting | Reported. A standardized extraction form was used, data were extracted independently by two reviewers, cross checked for consistency, and organized in Microsoft Excel. |
| Methods | 11 | Data items | List and define the variables for which data were sought and any assumptions or simplifications made. | Section 2.6 Data charting | Reported. Extracted variables included first author and year, clinical area, study design, population characteristics, assessed variables, and key findings. |
| Methods | 12 | Critical appraisal of individual sources | If critical appraisal was performed, provide the rationale and describe the methods used. | Section 2.7 Critical appraisal of individual sources | Not applicable. A formal risk of bias assessment was not performed, consistent with the mapping objective of the scoping review. The rationale is reported. |
| Methods | 13 | Synthesis of results | Describe how data were handled, summarized, and synthesized. | Section 2.8 Data synthesis | Reported. A qualitative thematic mapping approach was used, with studies grouped into OSA and frailty, OSA and sarcopenia, and sarcopenia and frailty in HNC. |
| Results | 14 | Selection of sources of evidence | Report numbers of records screened, assessed for eligibility, and included, with reasons for exclusion, preferably using a flow diagram. | Figure 1 and Section 3.1 | Reported. The PRISMA flowchart shows identification, screening, eligibility, exclusions, and final inclusion of 12 studies. |
| Results | 15 | Characteristics of sources of evidence | Present characteristics of each included source of evidence and provide citations. | Table 1 | Reported. Table 1 presents author, year, clinical area, study type, population, OSA assessment or case definition, assessed variables, key findings, and references. |
| Results | 16 | Critical appraisal within sources of evidence | If critical appraisal was performed, present the appraisal results. | Section 2.7 and Results | Not applicable. No formal critical appraisal was performed, and therefore no appraisal results are presented. |
| Results | 17 | Results of individual sources of evidence | For each included source, present the relevant charted data that relate to the review questions and objectives. | Table 1 and Sections 3.2 to 3.4 | Reported. Individual study findings are summarized in Table 1 and narratively synthesized in the Results. |
| Results | 18 | Synthesis of results | Summarize or present the charted results in relation to the review questions and objectives. | Sections 3.2 to 3.5 | Reported. Results are synthesized by thematic domain, and the evidence map distinguishes OSA related geriatric vulnerability from HNC related sarcopenia and frailty evidence. |
| Discussion | 19 | Summary of evidence | Summarize the main results, including key concepts, evidence types, and relevance to the review questions and intended audience. | Discussion, Sections 4.1 to 4.5 | Reported. The discussion summarizes evidence streams, biological plausibility, clinical relevance in HNC, contextual OSA and HNC linkage, and the integrated conceptual model. |
| Discussion | 20 | Limitations | Discuss limitations of the scoping review process and evidence base. | Section 6 Limitations of the Evidence | Reported. Limitations include limited number of studies, heterogeneity, observational design, lack of direct three way HNC evidence, variable assessment methods, and restricted search scope. |
| Discussion | 21 | Conclusions | Provide an interpretation of findings in relation to objectives and describe potential implications or next steps. | Section 8 Conclusions and Section 7 Future directions | Reported. Conclusions emphasize converging markers of reduced physiological reserve and the need for prospective studies integrating OSA, sarcopenia, and frailty assessment. |
| Funding | 22 | Funding | Describe funding sources for the scoping review and, where applicable, funding of included sources of evidence and the role of funders. | Funding statement | Reported for the present scoping review. The authors declare that no financial support was received for the research, authorship, or publication. Funding of individual included studies was not charted as a data item. |

Source guideline: Tricco AC, Lillie E, Zarin W, et al. PRISMA Extension for Scoping Reviews (PRISMA-ScR): Checklist and Explanation. Ann Intern Med. 2018;169(7):467-473. doi: 10.7326/M18-0850.
